# A Microfluidics and Agent-Based Modeling Framework for Investigating Spatial Organization in Bacterial Colonies: The Case of *Pseudomonas Aeruginosa* and H1-Type VI Secretion Interactions

**DOI:** 10.3389/fmicb.2018.00033

**Published:** 2018-02-06

**Authors:** Jared L. Wilmoth, Peter W. Doak, Andrea Timm, Michelle Halsted, John D. Anderson, Marta Ginovart, Clara Prats, Xavier Portell, Scott T. Retterer, Miguel Fuentes-Cabrera

**Affiliations:** ^1^Oak Ridge National Laboratory, Biosciences Division, Oak Ridge, TN, United States; ^2^Computational Sciences and Engineering Division, Oak Ridge, TN, United States; ^3^The Bredesen Center, University of Tennessee, Knoxville, TN, United States; ^4^Department of Mathematics, Universitat Politecnica de Catalunya, Barcelona, Spain; ^5^Department of Physics, Universitat Politecnica de Catalunya, Barcelona, Spain; ^6^School of Water, Energy and Environment, Cranfield University, Cranfield, United Kingdom; ^7^Computational Sciences and Engineering Division, Oak Ridge, TN, United States

**Keywords:** agent-based modeling, *Pseudomonas aeruginosa*, Type VI secretion, silicon microwell arrays, microbial succession, microbial organization, spatial confinement

## Abstract

The factors leading to changes in the organization of microbial assemblages at fine spatial scales are not well characterized or understood. However, they are expected to guide the succession of community development and function toward specific outcomes that could impact human health and the environment. In this study, we put forward a combined experimental and agent-based modeling framework and use it to interpret unique spatial organization patterns of H1-Type VI secretion system (T6SS) mutants of *P*. *aeruginosa* under spatial confinement. We find that key parameters, such as T6SS-mediated cell contact and lysis, spatial localization, relative species abundance, cell density and local concentrations of growth substrates and metabolites are influenced by spatial confinement. The model, written in the accessible programming language NetLogo, can be adapted to a variety of biological systems of interest and used to simulate experiments across a broad parameter space. It was implemented and run in a high-throughput mode by deploying it across multiple CPUs, with each simulation representing an individual well within a high-throughput microwell array experimental platform. The microfluidics and agent-based modeling framework we present in this paper provides an effective means by which to connect experimental studies in microbiology to model development. The work demonstrates progress in coupling experimental results to simulation while also highlighting potential sources of discrepancies between real-world experiments and idealized models.

## Introduction

Spatial organization has a strong influence on the development and dynamics of biological systems (Kreft et al., 1998; Lardon et al., 2011; Halsted et al., 2016; Hansen et al., 2016; McNally et al., 2017; Timm et al., 2017). The factors leading to changes in organization of multicellular assemblages at fine spatial scales are not well characterized or understood, however, they are expected to guide the succession of community development and function toward specific outcomes (Liu et al., 2009; Cline and Zak, 2015; Dini-Andreote et al., 2015). The organization of distinct microbial populations can be shaped by physical and chemical processes, and affect important activities such as antibiotic resistance, efficient energy conversion, C and N cycling and quorum sensing (Ginovart et al., 2005; Gras et al., 2010, 2011b; Sahari et al., 2014; Wang and Ma, 2014; Koonin and Wolf, 2015; Biteen et al., 2016). Microbe-microbe interactions can also depend on direct and indirect competition for resources between different community members (Kreft, 2004; Hellweger et al., 2008; Borenstein et al., 2015; McNally et al., 2017). The microscale/local transport of essential microbe-derived metabolites and cell-to-cell competition are likely to be strongly influenced by spatial confinement and individual cell behavior in the environment (Lardon et al., 2011; Pintelon et al., 2012; Vogel et al., 2015; McNally et al., 2017). Consequently, investigating the complexity of these processes and emergence of unique behaviors requires the combination of experimental and computational tools that can be used to explore the impact of spatial organization, while correlating individual microbial behavior and interactions to specific outcomes (Dini-Andreote et al., 2015; Zhu et al., 2015; Hansen et al., 2016).

Cells can compete directly with surrounding species through physical contact, and in more specialized cases, are capable of transferring toxic effector proteins to susceptible cells. The Type VI secretion system (T6SS) is an important example of such a pathway, being responsible for the assembly of a pilus apparatus that can be used to contact neighboring cells and potentially induce cell death (Hood et al., 2010; Chou et al., 2012; LeRoux et al., 2012). Hood et al. (2010) showed that the H1-T6SS of *Pseudomonas aeruginosa* is required to direct the injection of toxins from T6SS active cells (T6SS+) into T6SS-susceptible cells (T6SS−) that lack immunity. Other important secretion systems such as H2- and H3-T6SS in *P*. *aeruginosa* direct toxins preferentially to eukaryotic cells. However, because the H1-T6SS toxin is preferentially directed toward other bacteria, it is particularly well suited for studies of contact-mediated interactions between neighboring and competing prokaryotes (Mougous et al., 2006; Sana et al., 2016, 2017). T6SS interactions in mixed microbial populations also play an important role in the regulation of more complex biological processes and microbial community dynamics (Russell et al., 2014; Verster et al., 2017). For instance, the T6SS interactions occurring amongst commensal bacteria in the mammalian gut microbiome have been shown to modulate community composition and interactions, as well as provide a mechanism for defending commensal bacteria from invading pathogens (Hecht et al., 2016). Furthermore, these T6SS interactions are highly active and prevalent, where > 10^9^ T6SS firing events (i.e., predicted pilus injections) min^−1^ g^−1^ colonic contents can occur and nearly 25% of human gut microbiota have been shown to encode a T6SS pathway (Wexler et al., 2016; Sana et al., 2017).

Using two-member communities as a model system of T6SS interactions in the laboratory, Borenstein et al. (2015) demonstrated that established colonies of T6SS− *Escherichia coli* could survive contact with T6SS+ *Vibrio cholerae*. Agent-based modeling (ABM) simulations further showed that T6SS− cells could survive T6SS+ attack when placed in situations of nutrient limitation and relatively slow growth rates, and could even outcompete the T6SS+ cells, as long as T6SS− cells were able to establish microcolonies within the mixed community (Borenstein et al., 2015). These results demonstrate the importance of spatial confinement and local organization on cell growth and survival. Thus, competition between neighboring microbial cells and spatial confinement are expected to drive changes in cell assemblage and organization (Borenstein et al., 2015; Halsted et al., 2016; Hansen et al., 2016).

Numerous advances in our understanding of cell-to-cell behavior and interactions at fine spatial scales have stemmed from the use and development of nano/micro-fabricated platforms (Wang et al., 2013; Yamazaki et al., 2014; Swennenhuis et al., 2015; Xue et al., 2015; Hansen et al., 2016; Zhang et al., 2016; Timm et al., 2017; Yeh et al., 2017). Timm et al. (2017) used a microwell array platform to study the contact-mediated T6SS interactions of *P*. *aeruginosa*. The microwell array platform enabled high-resolution and high-throughput imaging of mixed T6SS+ and T6SS− cells growing under spatial confinement within microwells, with well diameters ranging from 20 to 100 μm and 5 μm depth. Interpreting the results of these cell-to-cell interactions with simplified analytical models of overall growth within each well becomes challenging and potentially unreliable when trying to capture the complex interactions reflected by spatial organization of microorganisms within the microwells. Alternatively, ABM simulations can capture how changes at the level of individual microbial interactions lead to changes observed at the community and microcolony levels. In conjunction with laboratory experiments ABM simulations can be used to infer and test important growth parameters that impact spatial organization within colonies (Borenstein et al., 2015).

In this study, we have developed an ABM model around experimental data obtained from a microwell array platform. We use the model to interpret spatial organization patterns of *P*. *aeruginosa* mutants growing under spatial confinement. The novelty of our approach relies on the high throughput nature of both the experiment and ABM simulations, which allows investigating how the initial ratio of community member abundances, initial growth location and T6SS interactions affect spatial organization during growth. The model is written in the language NetLogo (Wilensky(1999), NetLogo, http://ccl.northwestern.edu/netlogo/; Center for Connected Learning and Computer-Based Modeling, Northwestern University, Evanston, IL) and is linked to a computational framework that permits submitting many calculations in parallel for different initial parameters, where each combination of parameters can be conceptualized to represent a micro-environment of interest. The ABM model has been deployed in the Compute and Data Environment for Science, CADES (http://cades.ornl.gov/), which also stores the relevant experimental data used during fitting routines. We find that key parameters, such as spatial constraints, local concentrations of growth substrates/metabolites and associated rate constants alter the impact of *P*. *aeruginosa* Type VI secretion activity on the spatial organization of cells in confined environments.

## Materials and methods

### Bacterial cell culture

Two *P*. *aeruginosa* PAO1 mutants were modeled during growth simulations to investigate the effects of Type VI secretion on cell organization. Cultures included a ΔretS mutant that constitutively expresses GFP and the toxic effector proteins associated with Type VI secretion, and a ΔretS/Δtse/i1-6 deletion mutant that constitutively expresses m-Cherry and is susceptible to Type VI secretion interactions (i.e., injection of toxic effector proteins) (Timm et al., 2017). Cell culture conditions for growth experiments followed Hood et al. (2010) and Timm et al. (2017).

### Microwell fabrication

Fabrication of Si microwells followed the methods outlined in (Hansen et al., 2016). Briefly, a 1 μm parylene film was deposited on a 4 inch diameter silicon wafer with a silicon dioxide coating. An adhesion promoter and positive photoresist were spun onto the wafer with a spin coater, followed by 1 min of baking on a hot plate at 115°C. The substrate was exposed to UV light using a contact mask aligner, baked on a hot plate for an additional minute at 115°C, then developed. The parylene exposed in the patterned photoresist was etched with O_2_ plasma in a Reactive Ion Etch (RIE), and was followed by a Bosch process to etch into the silicon to form microwells. Residual photoresist was removed by etching with O_2_ plasma. The final well depth was 5 μm. The layout of the microwell platform can be found in Hansen et al. (2016). It contains arrays of microwells ranging in size from 5 to 50 μm in diameter, in increments of 5 microns, along with wells that are 100 μm in diameter. Twelve arrays of each size, including four replicates of three different well spacing, are included in each array. Individual microwell-array chips were sectioned from the Si wafers and subsequently used for growth experiments with *P*. *aeruginosa* mutants.

### Microwell culture experiments and image analysis

**Figure 1:**
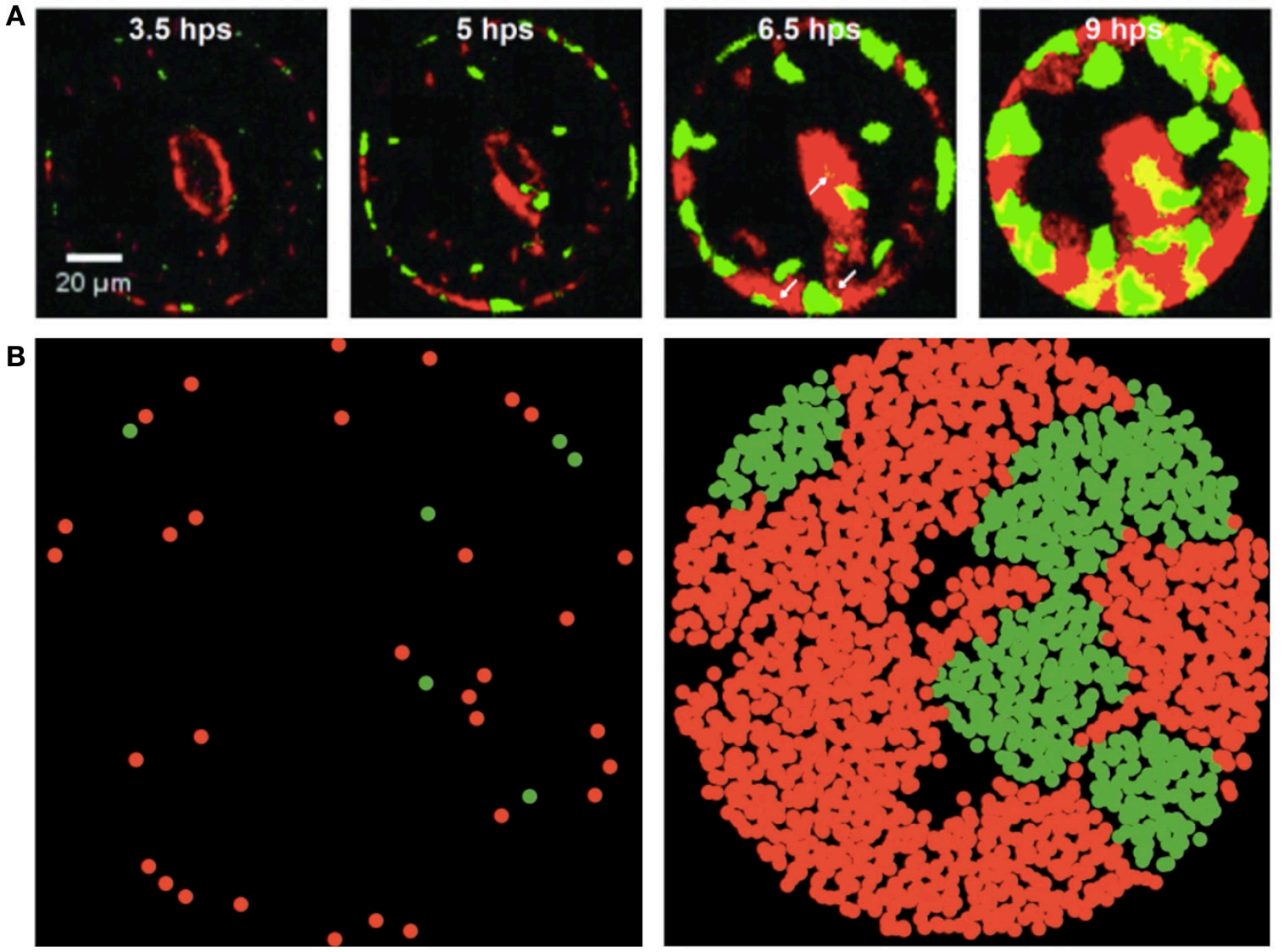
Experimental and simulated images of *P. aeruginosa* T6SS + and − mutants during growth in microwells (green and red fluorescence, respectively). **(A)** Mutants shown during growth in 100 μm well and images collected with a fluorescence microscope at 3.5, 5, 6.5 and 9 h post seeding (hps). Scale bar is 20 μm. Experimental images are those presented in Figure 6 of Timm et al. (2017) and reproduced here with permission. Further analytical details of how the images were acquired can be found in Timm et al. (2017). **(B)** Early and late stages of simulated growth using the agent-based model (left to right).

The cell-seeding, growth, imaging and image analysis methods used in this study correspond to those described in Timm et al. (2017). Timm et al. (2017) provides a detailed step-by-step description of those protocols along with online video content displaying those techniques. Representative data, as well as the image collection and correction procedures, are provided there. Specifically, we provide additional analysis of a more comprehensive data set and describe the development of a new ABM framework that helps explain some of the unresolved questions reported by Timm et al. (2017). Briefly, cells of *P*. *aeruginosa* were mixed in a 1:2 ratio (GFP:m-Cherry) suspended in growth media and incubated on bovine serum albumin (BSA)-functionalized microwell chips in a humid environment for 1 h (Timm et al., 2017). The number of cells that attach within any given well is dependent on the number of cells present in solution and the time allowed for the cells to attach. Not all of the cells will attach inside a microwell during a 1 h interval. Some cells remain in suspension or attach outside of the wells on the parylene cover. Following 1 h incubation the solution is removed and the parylene is peeled, leaving behind only cells that were attached inside the wells. The entire microwell array is then sealed under a nutrient agarose layer and grown in a live cell chamber on an automated microscope stage. Details of fluorescence microscopy used to measure growth are given in Timm et al. (2017). Images were collected every 30 min over a 24 h period, and were then processed and analyzed using ImageJ and Matlab software (Timm et al., 2017). Data presented here is taken from images collected from multiple chips imaged during experiments performed on different days. Experimental data for co-culture experiments represents analysis from 63 (20 μm), 35 (25 μm), 49 (30 μm), 16 (35 μm), 16 (40 μm), 16 (45 μm), 16 (50 μm), and 4 (100 μm) wells. Mono-culture experiments were performed as controls, with equivalent numbers of wells examined for T6SS+ and T6SS− only cultures at each well size. Representative images taken from a single 100 μm diameter well are shown in Figure 1A. Growth curves were established by measuring total fluorescence from both GFP and m-Cherry expressing mutants over time in each well and corrected for background fluorescence (Timm et al., 2017).

### Analysis of experimental imaging data

**Figure 2:**
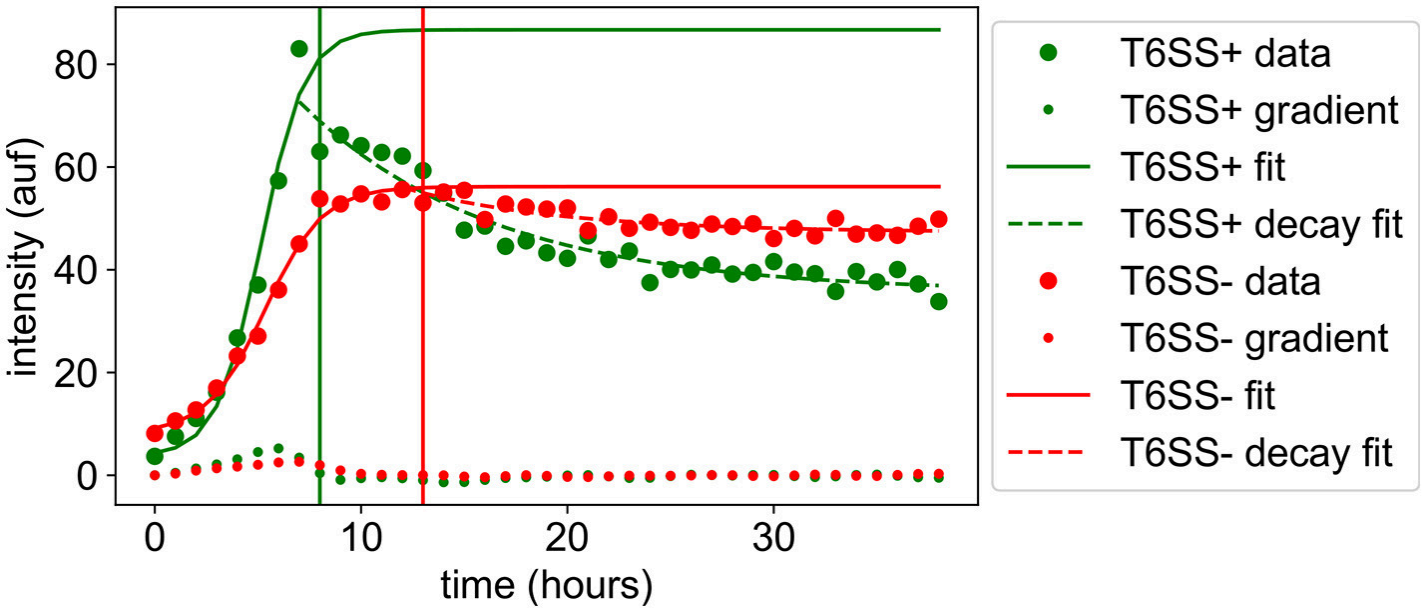
Growth trajectories of *P. aeruginosa* T6SS + and − mutants during growth in microwells (green and red lines, respectively). Experimental data from 30 μm wells are shown as closed circles. Solid lines show the logistic fit to the growth phase. Dotted lines show the fit of an exponential to the decay phase. The gradient zero crossing used as the division between the growth and decay phases is shown by a vertical line in the plot. Vertical y-axis values represent the relative microbial population (pop) abundances based on corrected fluorescence intensities and horizontal x-axis values represent time in hours. The notation auf stands for arbitrary units of fluorescence intensity.

A multi-stage fitting was performed on both the ABM population curves and time series fluorescence data corresponding to the experimental population curves of T6SS+ and T6SS− in each well. Using a derivative zero crossing algorithm, the growth period of each fluorescence series was separated from the decay stage (Figure 2). The data from the cell growth period was then fit to the logistic function, Equation 1, using the least squares Trust Region Reflective algorithm implemented in the Python scipy 0.19.0 library:

(1) $$\begin{array}{r} \frac{a}{1+e^{4}\frac{\mu }{a}\left(\tau -t\right)+2}+a_{0} \end{array}$$

where *a*_0_ is the initial fluorescence intensity, which reflects the number of living cells present inside the well at the beginning of the experiment. In this form, *a* is the maximum intensity, μ is the maximum growth rate and τ is the lag time. The analysis presented in this paper deals exclusively with observations of the growth phase. The parameters *a*, μ and τ for each well were collected and *a* and μ scaled as shown in (Eq. 2) to represent per unit intensity values; the scaled *a*′, μ′, and τ were then plotted vs. the initial ratio T6SS+:T6SS− for each well size and a linear fit was performed. The slope of each line, i.e., *a*′*/ratio*, μ′*/ratio* and τ*/ratio*, for each well was then plotted vs. all the well sizes.

(2) $$\begin{array}{r} a^{\prime }=\frac{a}{a_{0}}\\ \mu^{\prime }=\frac{\mu }{a_{0}} \end{array}$$

### ABM development and simulations

The model is described following the protocol ODD (Overview—Design Concepts—Details) that was initially established by Grimm et al. (2006) and later revised and updated by Grimm et al. (2010). This protocol was specifically developed in order to provide a standard way to describe ABMs, so that both the basic features and the details of the models could be correctly communicated to the scientific community. In the following, we provide a concise description of our particular ABM (see full details in the Supplementary text and Supplementary Figure 1 flowchart).

The basic principles of the bacterial model and protocol system developed here are taken from the INDISIM model (Ginovart et al., 2002; Gras and Ginovart, 2006; Gras et al., 2011a; Granda et al., 2016). The basic entities of our model represent bacterial cells of the two *P*. *aeruginosa* mutants, including the ΔretS mutant (T6SS+) and the ΔretS/Δtse/i1-6 deletion mutant (T6SS−) that is susceptible to Type VI secretion (Hood et al., 2010), and spatially confined areas (or grids) of a two-dimensional circular well that represents the growth environment on the microwell array chip used for laboratory experiments. The bacterial cells are defined by several individual variables and parameters: bacterial species (T6SS+ or T6SS−), mass, mass to initiate the division process, energy, and viability. Spatial grid variables contain the local content of a carbon (C) source, together with the x-y spatial coordinates. Global variables account for the global balance of bacteria (in terms of number and biomass for each of the two mutants of *P*. *aeruginosa*) and nutrient source, as well as the emerging bacterial and biomass mean growth rates, and the bacterial biomass distributions. The model can simulate a population of up to 10^5^ bacterial cells in spatial grid domain. This is a qualitative version of the model as it currently uses values that are given in relative units.

It is assumed that bacteria are able to respond to and detect the nutrient concentration in the space in which they are located. Both T6SS+ and T6SS− bacteria are modeled to consume resources at the same rate. The nutrient consumption is adjusted according to its local availability and the uptake is driven by the local concentration of available C. Cell movement and reproduction may also be driven by the occupation of the surrounding growth space. The mutants interact by direct cell-to-cell contact via the Type VI secretion system and, in this case, a T6SS+ can kill a contacted T6SS− cell. Additionally, indirect interaction can occur through the competition for available C and for the occupation of space. Stochasticity is introduced through setting initial individual locations at random by using a Gaussian distribution around an expected mean value (initial individual mass, mass to start the reproduction cycle, viability time and cell lysis when optimal conditions for cellular maintenance are not met). Random variation is applied to individuals within grid spaces to deal with the initiation of the reproduction cycle in each bacterium and with the change in location of cells. For instance, the model permits assigning a probability of initial growth at the edge of the well, a behavior that, as it will be discussed later, is observed experimentally. Moreover, in the predation process by T6SS+ cells, randomness is considered in the identification of a neighboring T6SS− cell. This randomness accounts for the uncertainty in these processes and reflects the high variety of mechanisms that underlie the variability observed in real systems. In order to avoid privileged first-acting bacteria in the model, the order of the bacteria to perform simulated actions is chosen randomly at each time step.

Overall, the execution of the model consists of four main parts: (1) initialization of the system, where the initial population of T6SS+ and T6SS− mutants are defined and distributed according to the user's input parameters, the spatial cells are set up with the corresponding initial amount of nutrient, and global variables are formally evaluated for the first time; (2) the core of the simulation, with the main loop where all the individual actions and environmental processes take place iteratively until the end of the simulation; (3) the output of results at the end of each time step, both graphical representation and numerical evaluation, as well as a final external text file with the simulation outcome for further analysis; (4) analysis of the results and comparison to experimental data.

The population curves obtained with the ABM model were fit using Equation 1 and scaled as in Equation 2, and the resultant parameters *a*′, μ′, τ plotted vs. the initial T6SS+:T6SS− ratio, similarly to what it was done with the experimental parameters. The ABM model, however, contains many input values that define a very large parameter space, where different results are obtained by using a different set of input values. In this paper, we have chosen to inspect a range of input values in order to examine how the level of aggressiveness of T6SS+ cells affect the outcomes of the simulations. Specifically, for each well and T6SS+:T6SS− initial ratio, 100 simulations were run, each corresponding to the same set of input values. To mimic the experimental conditions better, T6SS+ and T6SS− cells were initially constrained to areas corresponding to the well dimensions, and the bacteria were added in initial ratios and densities corresponding to those observed in the experimental wells of the same size.

## Results

Previously, Timm et al. (2017) demonstrated that the GFP ΔretS mutant T6SS+ and the m-Cherry ΔretS/Δtse/i1-6 deletion mutant T6SS− that is susceptible to Type VI secretion, formed discrete microcolony assemblages during co-growth in microwells. Observed well populations were heterogeneous (Figure 1A) with distinct assemblages of individual species forming across the wells. Instances of T6SS− microcolony formation were unexpected based on the hypothesis that T6SS+ cells would dominate each well environment due to their directed injection of toxic effector proteins into susceptible cells. In the present study, we used ABM simulations (Figure 1B) to examine how spatial confinement and Type VI secretion can lead to growth conditions that allow the formation of microcolony assemblages and enable susceptible T6SS− cells to persist.

**Figure 3:**
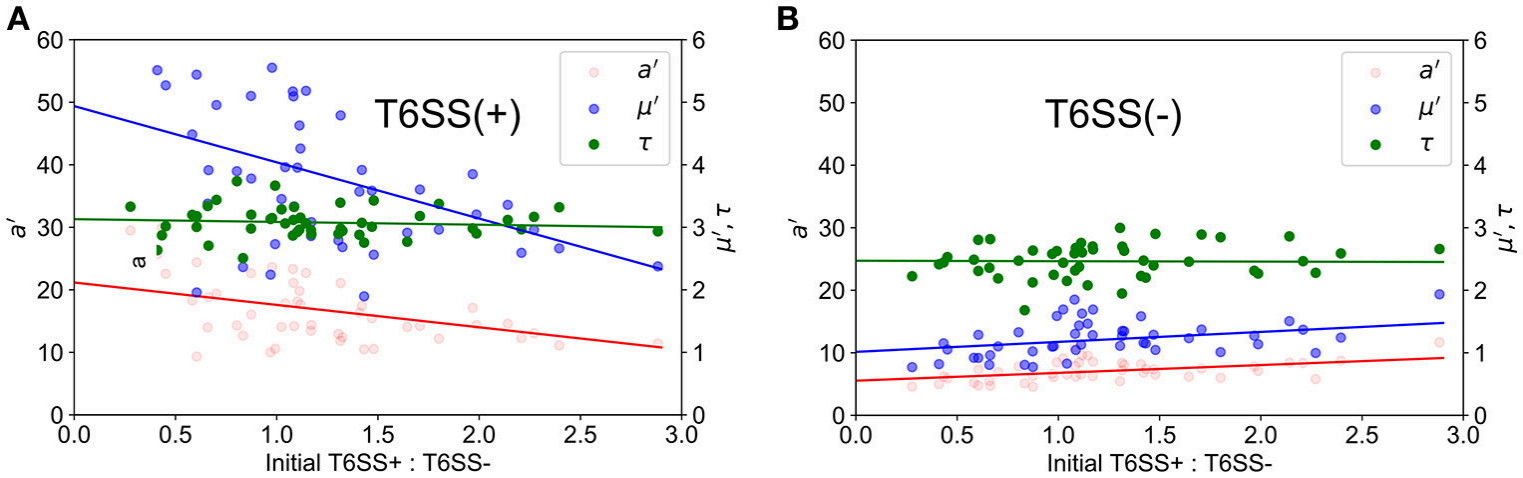
Experimental growth parameters vs. initial relative ratios of mutants. *a*′ (red), μ′ (blue) and τ (green) variables refer to maximum corrected fluorescence intensity (i.e., maximum relative cell abundance), maximum rate in change of fluorescence intensity (i.e., relative growth rate) and lag time to start of growth, respectively. Closed circles indicate corrected experimental data and lines represent regression trends. **(A)** T6SS+ growth parameters as a function of initial relative ratios of mutants when both mutants are grown together. **(B)** T6SS– growth parameters as a function of initial relative ratios of mutants when both mutants are grown together.

**Figure 4:**
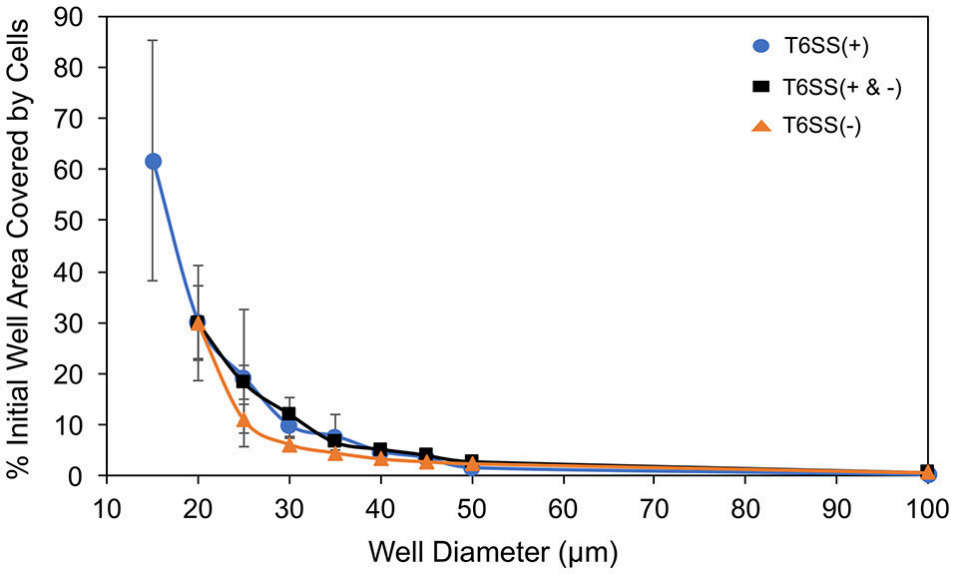
Well area (%) covered by initially seeded cells vs. well size. T6SS+ mutant only data (blue circle), combined mutant data (black square) and T6SS– mutant only data (orange triangle). The expected ratio of T6SS(+):(−) was 1:2. Error bars represent standard deviations around the mean value.

In Figure 3, we show how the experimental values for *a*′, μ′, τ change with the initial T6SS+: T6SS− seeding ratio for both types of cells in 30 μm diameter wells. Although the initial ratio of cells in the solution used to seed the wells is 1:2, natural variability in the number of cells of each type that seed into the wells provides initial ratios ranging between nearly 0 (all T6SS−) to 3:1 (3 times more T6SS+ than T6SS−). For both types of cells, τ is practically independent of the initial ratio, whereas *a*′ and μ′ for T6SS+ linearly decrease as the T6SS+:T6SS− ratio increases. Likewise, μ′ and *a*′ values decrease as cultures have an increasing number of T6SS− cells compared to T6SS+. For both T6SS− and T6SS+ the maximum number of cells per initial number of cells (*a*′*)*, as well as the maximum rate of increase per initial numbers of cells (μ′*)*, decreases as the corresponding cell type is initially present in excess. As seen in Figure 4, for each 30 μm well, the initial density is relatively constant, independent of the initial T6SS+:T6SS− ratio. Cells are, for the most part, segregated into domains that contain either T6SS + or − cells. The latter is observed both in experiment and simulations (Figures 1A,B, respectively), though some overlap can be seen within the experimental images (Figure 1A, yellow region). We speculate that larger clusters or colonies of cells will grow more slowly on a per cell basis because of nutrient limitations that may occur at the center of those microcolonies relative to growth at the edges. Thus, even though the overall density within the wells is the same, local microcolony size may influence the maximum growth rate per initial number of cells (μ′*)* and maximum number of cells per initial number of cells (*a*′*)*. A more detailed image analysis strategy that allows quantitative description of microcolony size and patchiness within wells throughout these experiments is warranted and under development.

During growth on the experimental microwell platform, both T6SS + and − cells showed a general preference for seeding at the well edges regardless of well size (Timm et al., 2017). During ABM-simulated growth in a circular well with preferential seeding at the boundaries, microcolony formation qualitatively mirrors the experimental results of Timm et al. (2017) (Figure 1) though, again, a more quantitative analysis of microcolony size and patchiness within wells is needed to make a direct comparison. In the simulations, the cell placement is biased so that there is a higher probability of cells beginning the simulation at the edges of a well. Cells seeded near the edge of wells, in both the experiments and the simulations, propagate toward the interior because of confinement imposed by the well edge. Based on the contact mediated pathogenesis associated with T6SS interactions, we expect that subsequent cell-to-cell interactions would help maintain bacterial domain segregation and minimize co-localization.

**Figure 5:**
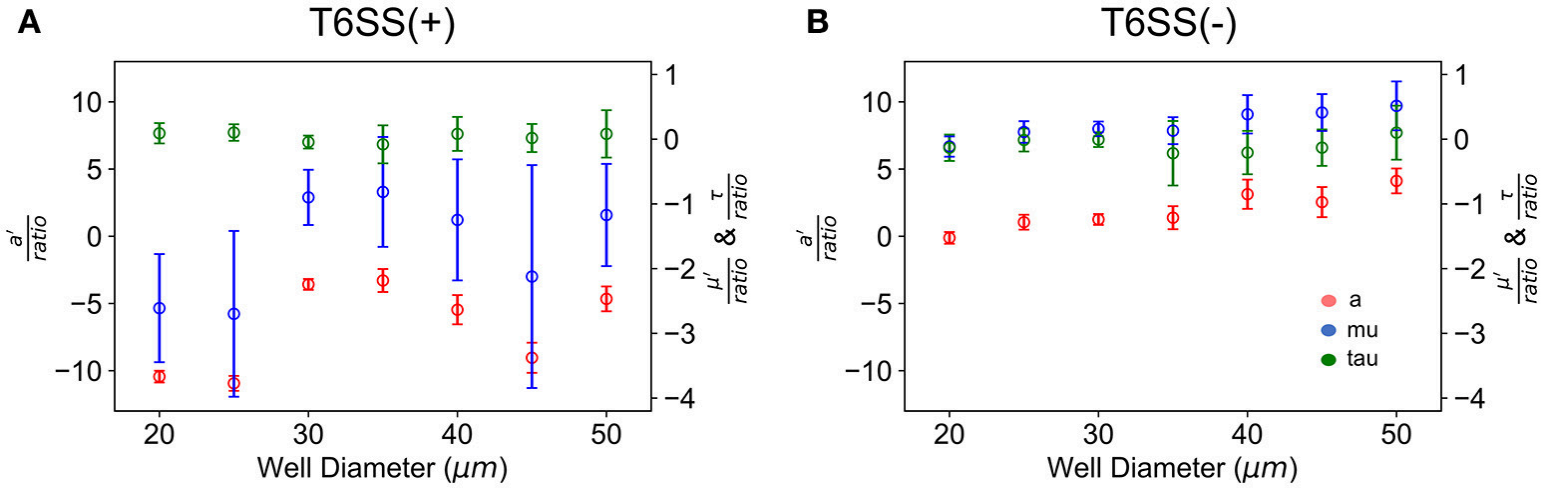
Growth parameters vs. well size. *a*′/initial T6SS+:T6SS– ratio (red), μ′/initial ratio (blue) and τ/initial ratio (green) are the slopes of the linear fit of the growth parameters with respect to the initial ratio as determine for each well size. **(A)** T6SS+ growth parameters as a function of well size when mutants are grown together. **(B)** T6SS– growth parameters as a function of well size when mutants are grown together. Error bars represent the 95% confidence interval for the linear fit demonstrated in Figure 3.

In Figure 5 we have plotted the slope of the straight lines, i.e., *a*′*/ratio*, μ′*/ratio* and τ*/ratio*, for each well size vs. the well diameter. In the case of T6SS+ cells, the slope of these lines shows more variation than that seen for T6SS− cells. The reason for this remains unclear and may result from T6SS+ cells growing outside the wells and outside the analytical region of interest (see Supplementary Movie 1). Also, fluctuations in GFP expression or loss of GFP intensity may contribute to this variation across experiments. For T6SS− cells, the sensitivity (slope, μ′/ratio and *a*′/ratio) of maximum growth rate per initial number of T6SS-cells and maximum number of cells per initial number of cells, increases slightly with well diameter. Figure 4 shows that the initial seeded cell density per well on the experimental platform decreased as a function of increasing well diameter between 15 and 100 μm. One might expect that at lower cell densities, the sensitivity to initial ratio of T6SS+ to T6SS− cells would decrease because lower densities should correspond to fewer T6SS+ to T6SS− interactions. However, if increasing microcolony size is suspected to cause reductions in μ′ and *a*′, as described above, larger wells could facilitate formation of these larger domains by (i) allowing unimpeded growth of microcolonies and (ii) increasing the possibility for the seeding of larger microbial aggregates or flocs from solution. Again, more detailed image analysis techniques should facilitate future investigations using the array platform.

**Figure 6:**
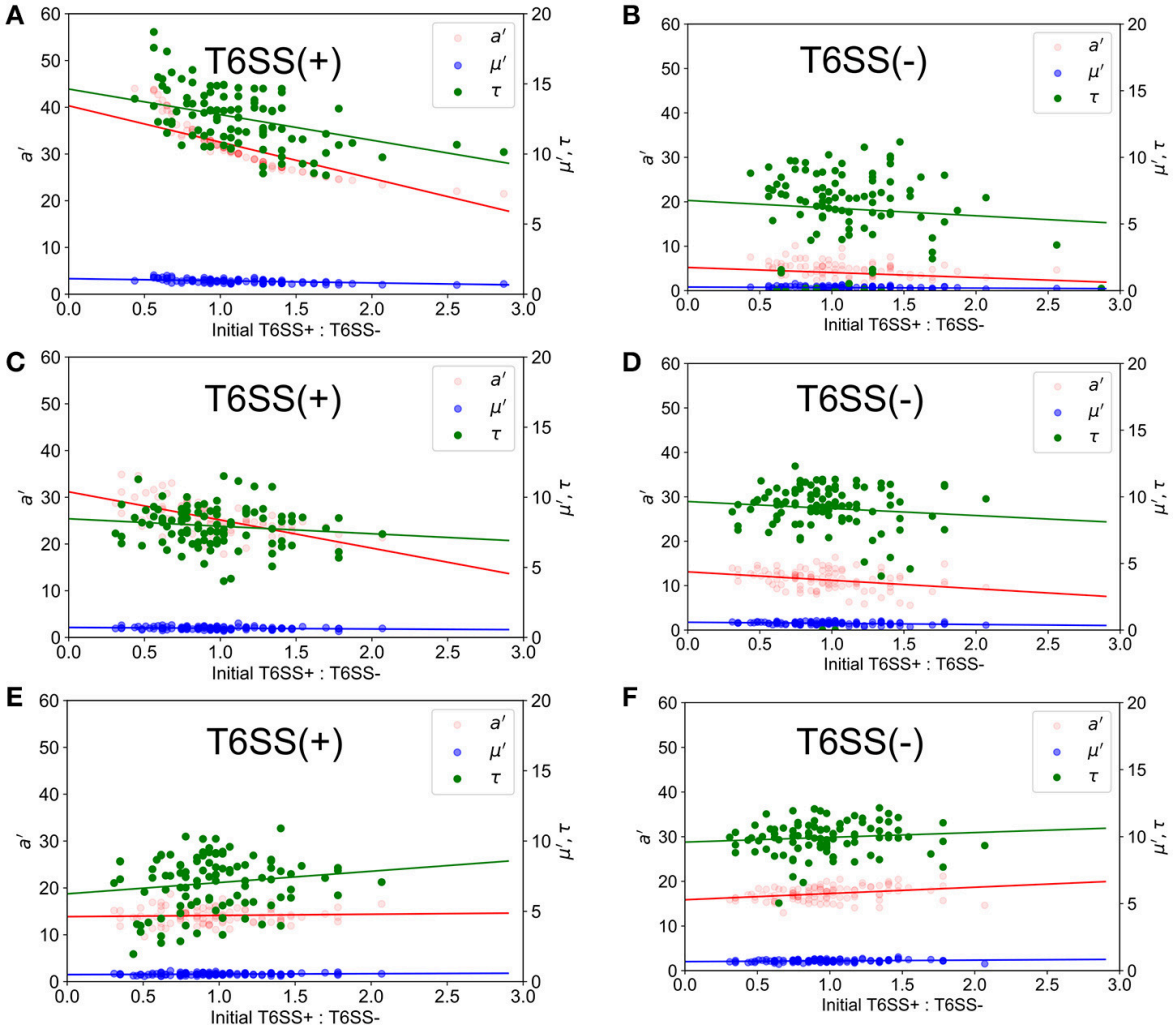
Simulated growth parameters *vs*. initial relative ratios of mutants. *a*′ (red), μ′ (blue) and τ (green) variables refer to maximum corrected fluorescence intensity (i.e., maximum relative cell abundance), maximum rate in change of fluorescence intensity (i.e., relative growth rate) and lag time to start of growth, respectively. Closed circles indicate ABM data and lines represent regression trends. (Left) T6SS+ growth parameters as a function of initial relative ratios of mutants when both mutants are grown together. (Right) T6SS– growth parameters as a function of initial relative ratios of mutants when both mutants are grown together. Aggressiveness of the T6SS+ in an immediate kill mode was set to a probability of kill on attack of 1.0 **(A,B)**, 0.1 **(C,D)**, and 0.01 **(E,F)**.

Using the ABM model, we performed a similar study of microcolony formation in a population of T6SS+ and T6SS− cells, where the aggressiveness of the former was changed from high to moderate to low. For simulating the experimental conditions closely, we computed the experimental distribution of T6SS+:T6SS− initial ratios and densities and used similar initial values in the simulations. The results of the simulations are shown in Figure 6 for a well size of 30 μm, where we have plotted *a*′, μ′ and τ for initial T6SS+:T6SS− ratios that resemble those obtained experimentally. When the aggressiveness was 1 (i.e., each contact occurring between a T6SS + and a T6SS - cell results in the death of a T6SS− cell), the behavior of *a*′, μ′ and τ for T6SS+ resembles that seen experimentally (compare Figure 6A to Figure 3A). The opposite is true for T6SS− cells (Figure 6B vs. Figure 3B), and the results indicate that the fewer T6SS− there are present, the more poorly T6SS− cells grow. The trend seen in Figure 6B for T6SS− cells can be explained as follows: while T6SS+ cells are not affected by their own aggressiveness, T6SS− cells are, and although it is theoretically advantageous for T6SS− cells to have relatively fewer neighboring T6SS− competitors, this advantage is offset by a high rate of lysis caused by the highly aggressive T6SS+ cells. Lowering the aggressiveness of T6SS+ from 100 to 10% does not significantly change the behavior of *a*′, μ′ and τ for both T6SS+ and T6SS− mutants (Figures 6C,D). However, if the aggressiveness is lowered to 1% (Figures 6E,F), then *a*′ and μ′ of T6SS− cells follow the same trend as in the experiment (Figure 3B). At this low killing rate, however, the growth rate of T6SS+ cells decreases (compare Figure 6E to Figure 3A). Specifically, as the aggressiveness of T6SS+ cells is reduced, the linear fit of *a*′ intersects the y-axis at a lower point. Thus, at lower abundances and lower levels of aggressiveness, T6SS+ cells can no longer effectively compete with T6SS− cells.

## Discussion

The T6SS of *P. aeruginosa* is an important biological model for understanding how cell-to-cell contact directs the succession and organization of microbial communities (Robinson et al., 2009; Hood et al., 2010; Sarris and Scoulica, 2011; LeRoux et al., 2012; Das et al., 2013). As mentioned in the introduction, T6SS interactions play a significant role in the regulation of microbiomes, which has important implications for biomedical and pathogen research, particularly for understanding mammalian gut microbiomes, and also environmental biogeochemistry relevant to native microbial interactions with plants and soil. However, the factors leading to changes in organization of microbial cells at fine spatial scales, driven by T6SS interactions, are not well characterized or understood. Recent laboratory investigations and ABM simulations indicated that established T6SS− colonies of *Escherichia coli* could persist during cell-to-cell interactions with *Vibrio cholerae* T6SS+ cells (Borenstein et al., 2015). The results of Timm et al. (2017) further suggested that spatial confinement, as well as T6SS activity between growing effector and susceptible *P. aeruginosa* mutants, could potentially direct cell organization in micro-colonies and affect the survival of susceptible cells. In the present study, building upon additional analysis of the complete dataset of Timm et al. (2017), and in combination with ABM simulations, we provide supporting evidence that both spatial confinement and T6SS activity can lead to changes in the organization and persistence of *P. aeruginosa*.

We found that discrete zones of clearing occurred around T6SS− cell assemblages during co-growth with T6SS+ cells in ABM simulations (Figure 1; see also Supplementary Movie 2). This cell-to-cell organization of T6SS− cells, surrounded by a zone of clearing, is consistent with T6SS-induced cell lysis at the boundary between both *P. aeruginosa* mutants (Hood et al., 2010; Borenstein et al., 2015). This zone of clearing provides a mechanism of *P. aeruginosa* cellular organization, as previously observed in Si-based microwell arrays (Timm et al., 2017). We speculate that during growth of both mutant strains, these buffer zones can occur randomly during growth, perhaps forming safe-pockets for susceptible cells to continue growing, and can become more defined as microcolonies of both species expand and interact at their outer boundaries. Fitting of the complete experimental dataset indicated that starting at the apparent peak in cell growth for both strains, a general decay in T6SS+ GFP signal intensities began, while T6SS− m-Cherry intensities subsequently remained more persistent over time (Figure 2). Borenstein et al. (2015) demonstrated that more-established microcolonies of T6SS-susceptible cells can potentially survive T6SS attack, which helps explain the persistence of susceptible *P. aeruginosa* mutants as deduced from the fluorescence intensities taken from our experimental data. We also observed that T6SS+ cells could outgrow a well once the interior of the well cavity had become nearly filled by growing cells (Supplementary Movie 1); this may explain, to some extent, the sharp decay phase generally observed for T6SS+ GFP intensities (Figure 2).

We found that the initial seeded cell density per well on the experimental platform decreased as a function of increasing well diameter between 15 and 100 μm (Figure 4), but cell density did not have an apparent effect on cell organization during different growth simulations, which is consistent with the results of Timm et al. (2017) that demonstrated microcolony formation across all well sizes between 20 and 100 μm diameters. The correlation between initial cell density after seeding and well size likely reflects the preparation of the experimental microwell platform. For instance, following the experimental cell seeding step (Timm et al., 2017): (1) slight drying of the aqueous culture media before contact with the nutrient agarose cover; (2) difficulty rinsing cells from smaller diameter wells during the final water rinse step; or (3) a larger side-wall to floor area ratio per well could have affected initial cell densities such that smaller wells were more densely packed than larger wells, particularly at well edge boundaries. However, qualitatively, we found that spatial organization into distinct T6SS mutant assemblages during experimental and simulated growth was not strongly influenced by cell density or close packing. Future quantitative analysis of assemblage size and spacing for different well sizes may reveal a more defined mechanism. Densely packed cell assemblages have been shown in previous studies to follow similar biological-phase separation where distinct microcolony formation is favored regardless of cell-to-cell density in spatially confined environments (Tolker-Nielsen and Molin, 2000; Berk et al., 2011; Borenstein et al., 2015; Cutler et al., 2015; McNally et al., 2017).

We generally found the impact of well size to be negligible for the size ranges explored in experiments, see Figures 5A,B. Well size did not have a significant impact on overall growth rates per initial cell number or maximum growth rate per initial cell number. This was unexpected. Indeed, in well diameters < 25 μm, competition for resources and cell-to-cell interactions would have been expected to suppress T6SS− growth. Reductions in spatial confinement within larger wells would, in principle, allow T6SS− cells to grow more efficiently with increasing well diameters, reducing the likelihood of encountering T6SS+ because of the lower seeding densities and more available area. In wells of 45 μm diameter and greater, at much lower initial densities (Figure 4), the individual T6SS− colonies may have had the potential to develop with less competition and become more established before interacting with the more aggressive mutant strain. In this case, the perimeter of T6SS-interactions around a colony would be overshadowed by the more established interior of each mutant colony. In other words, with larger wells above 40 microns, T6SS-killing should have become secondary to the size of mutant colonies by the time they interact at their edges. Clearly, a more systematic study of micro-colony size and distribution is needed to understand these results. The behavior of T6SS+ cells across well sizes displays variation in the data that makes it difficult to draw specific conclusions about T6SS+ growth as a function of well size.

**Figure 7:**
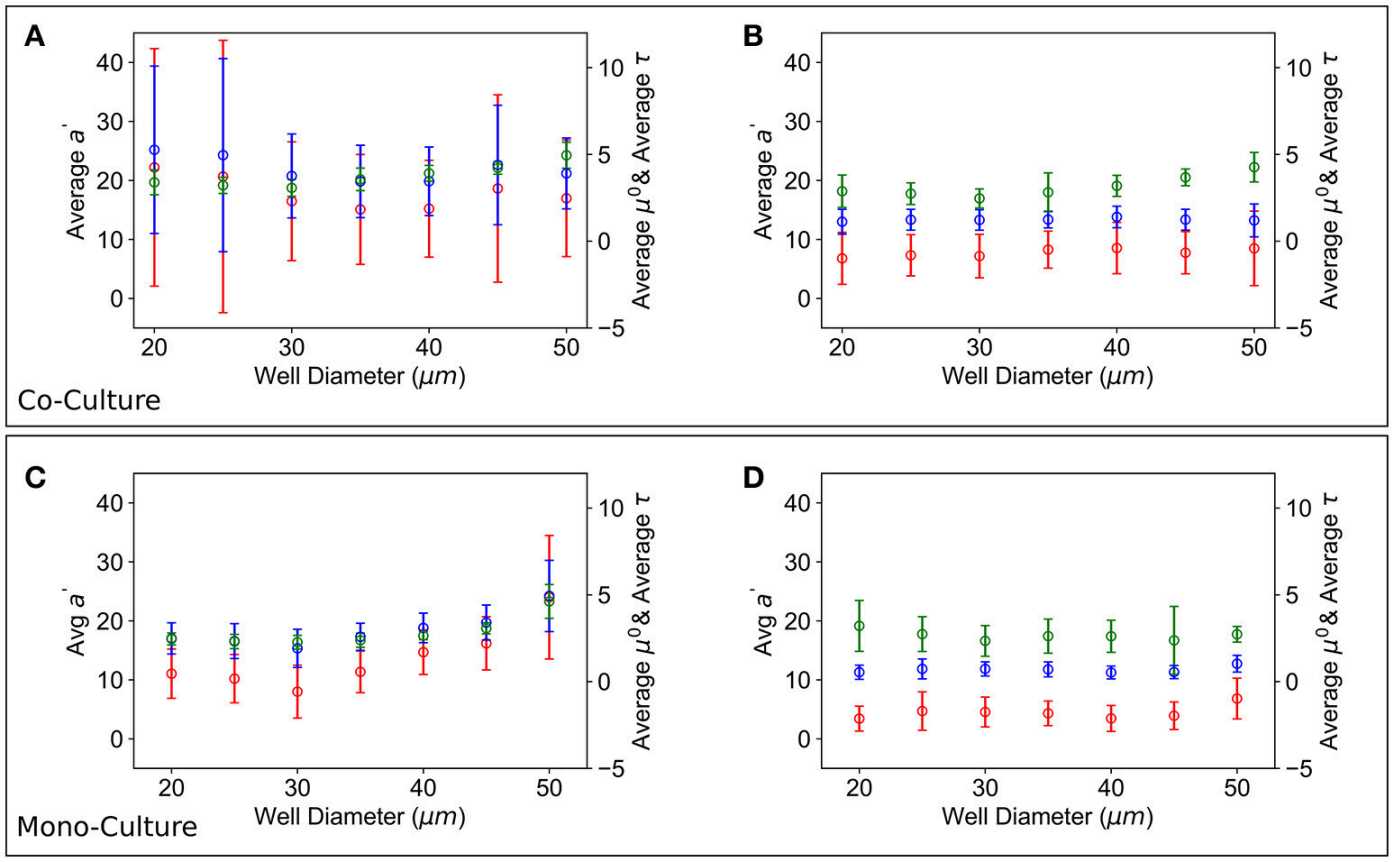
Growth parameters vs. well size. Average *a*′ (red), μ′ (blue) and τ (green) growth parameters over all the T6SS+:T6SS– initial ratios across wells of different size. **(A)** T6SS+ average growth parameters as a function of well size when mutants are grown together. **(B)** T6SS– average growth parameters as a function of well size when mutants are grown together. Error bars represent the 95% confidence interval for the linear fit demonstrated in Figure 3. **(C,D)** Panels show equivalent parameters in monoculture.

Finally, average *a*′, μ′ and τ for each well size vs. the entire well size distribution were also calculated, see Figure 7; the results obtained in a mixed population of T6SS+ and T6SS− cells were compared to those obtained in control experiments comprising only one cell type. As seen in Figure 7D, for the control experiments, the average *a*′, μ′ and τ of T6SS− cells are practically insensitive to the well size, whereas the same data for T6SS+, Figure 7C, shows variation and an increase at larger well sizes. In mixed populations, the data for T6SS− cells shows a similar trend, Figure 7B, although the error bars are larger, illustrating the interactions with T6SS+ cells. The data for T6SS+ cells, Figure 7A, shows even larger error bars, which is surprising because these cells should not be negatively impacted by the presence of T6SS− strains. As mentioned above, inspection of the data reveals that on some occasions T6SS+ cells can outgrow/leave the well boundaries (Supplementary Movie 1). We believe this is one of the primary reasons for the large variation observed in Figures 7A,B. Consequently, whether or not T6SS+ cells outgrow or escape the wells should also affect the growth of T6SS− cells remaining within the same wells.

In this study we have developed an experimental-ABM framework that can be used to interpret unique spatial-organization patterns of *P*. *aeruginosa* cells growing under spatial confinement. The ABM model developed here, although qualitative, is capable of showing microcolony formation regardless of initial density, or whether the bacteria prefer to begin growth at the well edges, which is consistent with other recent studies that have examined different microbial species under T6SS interactions. Our model was also capable of reproducing the behavior of *a*′, μ′ and τ of T6SS+ cells for a particular well size, and the same was true for T6SS− cells once the aggressiveness level of T6SS+ cells was lowered. As such, this model can be used to extract information regarding aggressiveness levels, amount of available resources, and rate of consumption of nutrients, as well as how all these variables affect the growth of the bacterial colony. Yet, there are some uncertainties that the current model does not take into account. Future work will focus on optimizing the model by identifying the most essential growth parameters and developing a more quantitative description of variables used for running the simulations. Further, the ABM model is capable of investigating 5000 wells in 30 min, and in connection with the microfluidic platform, constitutes a powerful framework to connect microbiological experiments to ABM simulations, while improving the ABM models to more accurately reproduce the experimental observations. Finally, this new microfluidic-ABM framework could be used in the future to predict the types of microcolonies that are likely to develop when different microbial species are mixed, which is expected to advance our understanding of microbial ecology at fine spatial scales, as well as mechanistically describe how microbial succession occurs in nature and shapes environments of interest.

## Author contributions

All authors were involved in the writing and proofing of the manuscript. JW, AT, MH, and SR were responsible for the design, execution and interpretation of the experimental platform and ABM results. PD, JDA, MG, CP, XP, and MF-C were responsible for implementing the ABM model, doing the simulations and interpreting the results.

### Conflict of interest statement

The authors declare that the research was conducted in the absence of any commercial or financial relationships that could be construed as a potential conflict of interest.

## Supplementary material

Supplementary Figure 1 Flow chart of the bacterial ABM model designed and implemented in the NetLogo platform.

Supplementary Movie 1 Co-growth of T6SS+ and T6SS- in the micro-fluidics device.

Supplementary Movie 2 ABM simulation of T6SS+ and T6SS- cells co-growth.
