# Supplementary figures and images for: A Microfluidics and Agent-Based Modeling Framework for Investigating Spatial Organization in Bacterial Colonies: The Case of Pseudomonas Aeruginosa and H1-Type VI Secretion Interactions
Source: Front Microbiol. 2018 Feb 6;9:33. doi: 10.3389/fmicb.2018.00033 (PMC5808251; doi:10.3389/fmicb.2018.00033)

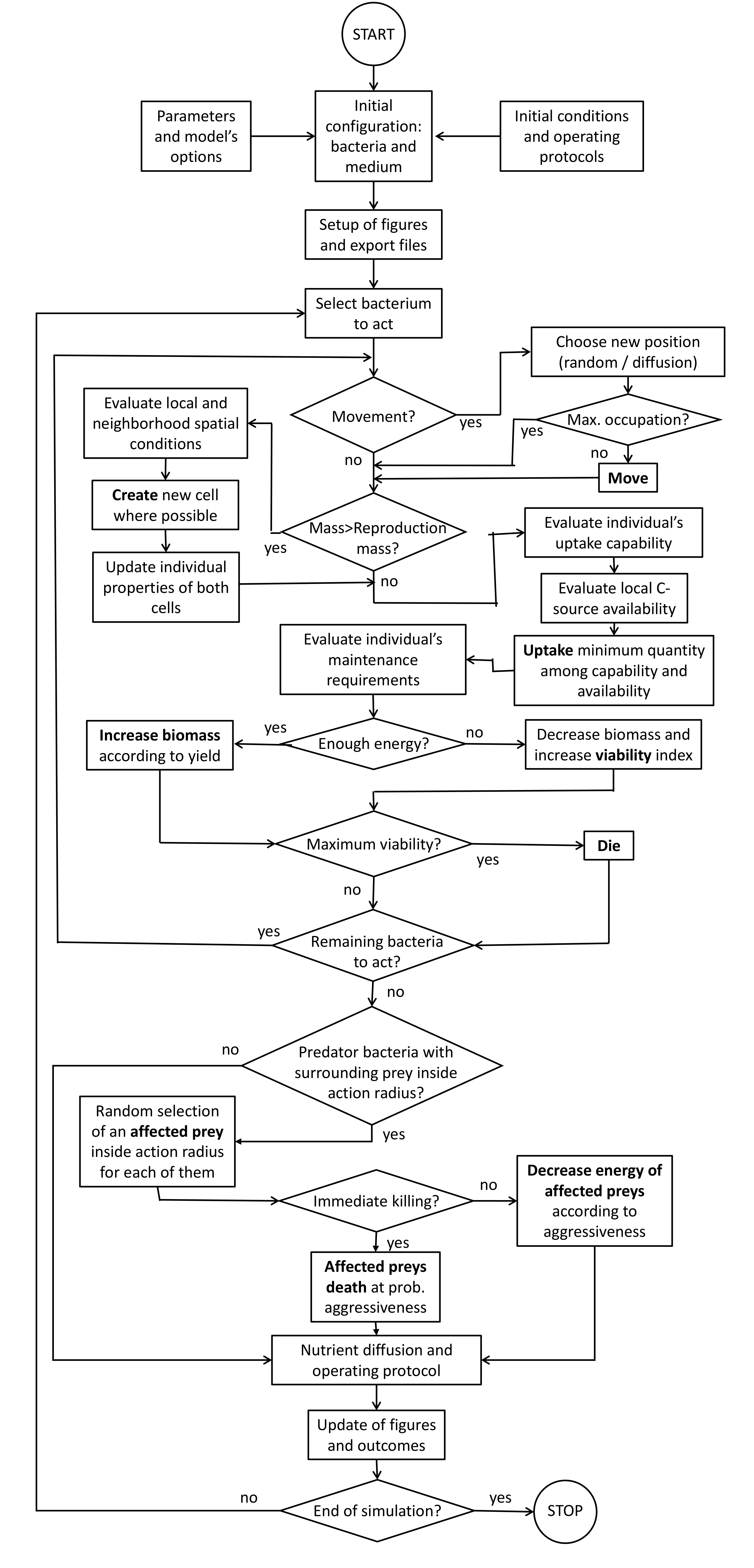

Supplement: Supplementary Figure 1 — Flow chart of the bacterial ABM model designed and implemented in the NetLogo platform. [file Image1.TIF]
