# Supplementary material for: A Microfluidics and Agent-Based Modeling Framework for Investigating Spatial Organization in Bacterial Colonies: The Case of Pseudomonas Aeruginosa and H1-Type VI Secretion Interactions
Source: Front Microbiol. 2018 Feb 6;9:33. doi: 10.3389/fmicb.2018.00033 (PMC5808251; doi:10.3389/fmicb.2018.00033)
Supplement: Supplementary file 4 [file Presentation1.PDF]

Supporting Information

A microfluidics and agent-based modeling framework for investigating spatial organization in bacterial colonies: the case of *Pseudomonas aeruginosa* and Type VI Secretion Interactions

Running Title: *Pseudomonas* Confinement and Type VI Secretion

Authors: Jared Wilmoth[1], Peter Doak[2], Andrea Timm[1], Michelle Halsted[3], John D. Anderson[3], Marta Ginovart[4], Clara Prats[5], Xavier Portell-Canal[6], Scott T. Retterer*[1,2] and Miguel Fuentes-Cabrera*[2,7]

[1]Biosciences Division, Oak Ridge National Laboratory, Oak Ridge, TN, United States,

[2]Center for Nanophase Materials Sciences, Oak Ridge National Laboratory, Oak Ridge, TN, United States

[3]University of Tennessee, Knoxville, TN, United States,

[4]Department of Mathematics, Universitat Politecnica de Catalunya, Castelldefels, Barcelona, Spain,

[5]Applied Mathematics Department and School of Agricultural Engineering, Universitat Politecnica de Catalunya, Castelldefels, Barcelona, Spain,

[6]Cranfield Soil and Agrifood Institute, Cranfield University, Bedfordshire, United Kingdom

[7]Computational Sciences and Engineering Division, Oak Ridge, TN, United States

1. ODD DESCRIPTION OF THE AGENT-BASED MODEL

The model is described following the protocol ODD (Overview – Design Concepts – Details) that was initially established by Grimm et al. (2006) and later revised and updated by Grimm et al. (2010). This protocol was particularly developed in order to provide a standard way to describe Agent-based Models (ABMs), so that both the basic features and the details of the ABMs could be correctly communicated to the scientific community.

1.1. Overview

Purpose

The purpose of this model is to provide a two-species basic bioreactor for investigating microbial interactions in communities consisting of predator and prey

bacteria. It is a model that can be used as a starting point for the approximate representation of the observed laboratory microbe array experiments.

Entities, state variables and scales

The basic entities of this model are individuals that represent bacteria, Predator and Prey, and spatial cells or grid cells of a two-dimensional lattice that represent the culture medium. Bacteria are defined by several individual variables and parameters: species (Predator or Prey, T6SS+ and T6SS- cells, respectively, in the manuscript), mass, mass to initiate the division process, energy index, and viability index. Spatial cell variables comprise the local content of a nutrient source, which is a C source, together with the x-y spatial coordinates. Global variables account for the balance of bacteria (in terms of number and biomass for each of the two species) and nutrient (C source), as well as the emerging bacterial and biomass mean growth rates, and the bacterial biomass distributions. The model can simulate a population of up to 10^5 bacterial cells in an IQxIQ spatial cell domain (IQ can be initially fixed). This is a non-parameterized version, so all the values are given in relative/qualitative simulation units. The values of the parameters are fixed and those values used in simulations are shown in Table 1.

Process overview and scheduling

The behavior of bacteria in the spatial domain is driven by a set of rules or sub-models that describe the following actions: movement (either random or random conditioned by diffusion); reproduction by bipartition; nutrient uptake of C source; metabolism with cellular maintenance and synthesis of new mass; and viability and death (due to starvation or to an external event). In addition, the possibility that one of the species (predator) may destroy the other (prey) is offered in two ways: a slow-progressive or immediate predation. At each time step of the simulation, bacterial cells perform the set of stated actions. Sub-models regarding spatial cells include the diffusion of nutrients and, if fed-batch protocol is chosen, the entrance of fresh medium according to the corresponding operating protocol. The occupation by bacteria of the bi-dimensional space is also controlled. These actions are performed once all the individuals have acted.

Overall, the execution of the model consists of four main parts: (1) initialization of the system, where the initial population of predator and prey are defined and distributed

according to the user's choices, the spatial cells are set up with the corresponding initial amount of nutrient, and global variables are formally evaluated for the first time; (2) the core of the simulation, with the main loop where all the individual actions and environmental processes take place iteratively until the end of the simulation; (3) the output of results at the end of each time step, both graphical representation and numerical evaluation, as well as a final external text file with the simulation outcome for further analysis; (4) analysis of the results and comparison to experimental data. Simulations were run for conditions that matched the experimental conditions. Specifically, the bacteria were mixed in initial ratios showing the same distribution of initial ratios as produced by the 1:2 seeding used in experiment with well sizes and densities in the wells following those observed experimentally. Bacterial uptake is described locally: the individual actions that occur only affect the spatial cell in which each bacterium is found, and the variable that controls the amount of nutrient for each cell is updated at the time its value is modified. The other individual actions, such as movement or predation, can be performed in the radius explicitly defined for those parameters. The occupation of the cells by the bacteria is asynchronously updated each time step.

1.2. Design concepts

Basic principles

The basic principles of the bacterial model and protocol system are taken from the IbM INDISIM (Ginovart et al., 2002, 2012).

Emergence

The global dynamics of the system is expected to emerge from the kind of interaction between both species combined with the initial conditions. The overall behavior of the population emerges from local nutrient concentration and individual bacterial behavior at each step of time.

Sensing

Bacterial cells are sensitive to their local environmental conditions. Although the product detection mechanism has not been explicitly modeled, it is assumed that individuals are able to perceive the nutrient concentration in the cell in which they are

located. As a consequence, the nutrient consumption is adjusted according to its local availability: the uptake is driven by the local concentration of C source. On the other hand, the movement or reproduction may be also driven by the occupation of the spatial cells. It is also assumed that individuals of species predators can perceive whether the cells around them are occupied or not by individuals of species preys, and identify cells to be destroyed.

Interaction

Individuals interact either directly or indirectly. They interact directly through the predation process: a predator can attack a neighboring prey. Indirect interaction occurs through the competition for the nutrient and for the occupation of space.

Stochasticity

Stochasticity is introduced when setting initial individual locations randomly, and some characteristics of individuals, using a Gaussian distribution around an expected mean value (initial individual masses, masses to start the reproduction cycle, viability time and lysis when optima conditions for cellular maintenance are not met). The Gaussian or normal distribution reflects range in the population and is usually met in nature and statistics (with a coefficient of variation, CV, which fix the variability of those distributions). Randomness is also considered when the rules are applied to individuals and to spatial cells to deal with the initiation of the reproduction cycle in each bacterium, with the change in location in the bacterial movement or for the assignment of a position for a new individual appearing in the reproduction process, and for the assignment of C source to be uptaken by the bacterium. Moreover, in the predation process, randomness is considered in the identification of a prey among the set of possible victims in the neighborhood of the predator. This represents the uncertainty in these processes and reflects the high variety of mechanisms that underlie the variability observed in real systems. In order to avoid privileged first-acting bacteria, at each time step the order of the bacteria to perform the actions is chosen randomly.

Observation

The user can follow the dynamics of the system by means of the observation of global variables in monitors or plots, both at the macroscopic level (bacterial concentration and biomass for each of the species, C source nutrient of the system, growth rates in

bacteria concentration and in biomass) and at the mesoscopic level (biomass distribution among the two populations). The evolution of the spatial distribution of nutrient source and bacteria distinguishing both species in the domain that represent the environment is also observable, as it is the distribution of the bacterial density of occupation of the space.

1.3. Details

Initialization

The initialization consists on the explicit setup of the system according to the initial, model and operating parameters chosen by the user. These are the main parameters (Table 1) to be defined:

i) Parameters of the system related to initial conditions: size of the bidimensional domain ("square-cell-dimension"); whether circular confinement is approximated by masking out spatial cells falling outside of a diameter "square-cell-dimension" circle ("circle-world"); the probability a bacterium is initially located at the edge of the world ("edge-prob"); if "circle-world" is true; initial number of bacteria for each of the two species ("initial_predator" and "initial_prey"); initial concentration of nutrient ("initial_local_C_source"); and the coefficient of variation for the Gaussian probability distributions used in the model ("CV") which reflects the variability assumed in the actions performed and in the distribution of characteristics or properties articulated by normal variables used in the virtual system.

ii) Parameters related with the operating system: type of movement for the individuals ("movement-type") depending on the conditions of the medium; protocol for the alimentation of the system ("protocol-of-feed"); and for fed-batch protocol, the local input quantity of C source ("fed_C_source") and frequency of this input ("length_time_feed") can be fixed. In a batch protocol, no entrance nor exit of individuals and nutrient take place.

iii) Parameters related with the bacterial model: the energy that a unit of C source uptaken supplies to the bacterium ("Yield_C_source"); constants that determine the availability of nutrient presents in the spatial cell and the uptake ratio of C source by a bacterium by time step ("Availability" and "Uptake_of_C_source"); the units of energy that a unit of mass of a bacterium need for the correct maintenance in each time step

("maintenance"); the maximum radius in the movement of the individuals ("radius-movement"); the maximum radius that determine the area where a predator can identify a prey ("radius-action-predator"); if predation action is immediate ("immediate-killing?"); the aggressiveness of the predator, which "if immediate-killing?" is true is the probability of prey death on a predator attack or which "if immediate-killing?" is false is the speed at which energy is removed from the prey ("aggressiveness"); the number of time steps that a damaged/non-viable cell can survive until its death (max_viability_time"); the mass to initiate the reproduction cycle ("max_reproduction") and in a self-degradation biomass process to supply energy for maintenance; the minimum mass that a bacterium can achieved ("min-mass").

iv) Parameters related with the medium model: the diffusion coefficient ("diff-coeff") for the nutrient and the maximum capacity of each spatial cell ("max-occupation") to harbor individuals.

The following switches can be used in order to fix some of the processes that will be taken into account during the simulation:

- Handling protocol for the medium: Batch (i.e., fixed amount of nutrient) / Fed-batch (i.e., periodic entrance of fresh medium)

- Bacterial motion type: None / Random / Random according to the diffusion coefficient

- Inter-species damage: immediate (On) or progressive (Off) predation of a prey.

Once all the parameters and the options have been chosen by the user, the explicit setup will generate the initial configuration of the system and the simulation will be ready to be run.

Input data

The model does not use any external input data.

Sub-models

1) *Movement.* The position of the bacterium is defined by two Cartesian coordinates. It is assumed that the position of a bacterium might change to a new position in the space.

The bacteriun position after the movement is randomly chosen in the circle determined by the radius of movement from its own position, as long as this new position belongs a spatial cell with an occupation below the maximum capacity fixed.

2) *Reproduction*. A bacterium's split_mass is assigned from a non-negative random draw from a normal distribution with mean "max_reproduction" and standard deviation equal to "CV"·"max_reproduction" each time a bacterium is created. A bacterium that has reached a mass above its split_mass will split into two bacteria if there is occupational vacancy in its spatial cell or any of its 4 nearest neighbor spatial cells. To this end, the bacterium's mass is halved and a new bacterium of the same species (predator or prey) and with the same mass is created in the current spatial cell if the number of bacteria in this patch is below the maximum occupation. Otherwise, a new random location is assigned in a nearest neighbor spatial cell with vacancy. If no free space exist in this time step the reproduction will not be accomplished and it will wait for the next time step.

3) *Uptake*. C source in the liquid phase of the medium can be taken up by bacteria during maintenance and growth. For each bacterium, the maximum individual uptake, in a time step will be determined by the multiplication of the size of the individual (i.e. its mass) and a uptake coefficient, a non-negative random draw from a normal distribution with mean "Uptake_of_C_source" and standard deviation equal to "CV"·"Uptake_of_C_source". The available amount of the C source is determined by multiplying its availability proportion with the current amount in the spatial cell where the bacterium is located. The final individual nutrient uptaken is below or equal to this available amount of the C source.

4) *Metabolism*. The amount of nutrient uptaken provides energy to the bacterium according to the value of the yield of C source assigned to each unit of this nutrient, and this energy is accumulated to the energy that a bacterium had. But, because a bacterium requires energy for its cellular maintenance, it is assumed that this energy is proportional to its mass. To cover this maintenance, the energy achieved will be decreased according to this requirement. If the bacterium has enough energy to cover the maintenance, it remains viable, and the half of the remaining energy will be used

to create new mass (1 unit of energy will be 1 unit of mass). If energy is not enough for maintenance and it mass is not under the minimum mass, it uses a portion (10 %) of its own mass.

5) *Viability and death*. Whenever a bacterium is not able to satisfy its maintenance requirements in a time step, the viability index of the bacterium increases by one. This index is evaluated by comparing this value with a non-negative random draw from an normal distribution of mean "Max_viability_time" and standard deviation equal to "CV"·" Max_viability_time", a time beyond which the bacterium cannot survive and dies.

6) *Predation*. A predator randomly identifies a prey within its area of predation, a circle centered on itself and radius "radius-action-predator". Once the prey is identified, the predator could immediately kill this prey with probability "aggressiveness", or it can damage it more or less aggressively, decreasing a higher or lower percentage of its energy. This progressive damage eventually leads to a fatal decrease of energy in the prey, causing it do die.

7) *Nutrient diffusion:* A periodic diffusion of nutrient, the C source, is performed in order that spatial differences in concentrations are smoothed.

8) *Entrance of fresh medium:* the input of C source is carried out according to the handling instructions given by the user at the beginning of the simulation. When a specific amount of nutrient has to be introduced, it is homogenously distributed among the spatial cells.

Table 1. Models parameters and their range.

**Initial system's parameters**	**Final ranges of values**	Possible Values
Square-cell-dimension	20-50 step 5	Positive float
Initial_predator	Determined from density and random ratio	Positive integer
Initial_prey	Determined from density and random ratio	Positive integer

Initial_local_C_source	5100	Positive float
CV	10	Positive float
Movement-type	Random	Null, Random, Random-diffusion
Protocol-of-feed	Batch	Batch, Fed-batch
Fed_C_source	N/A	Positive float
Length_time_feed	N/A	Positive integer
Circle-world	true	Boolean
Edge-probability	0.85	0-1
**Bacterial parameters**		
Yield_C_source	0.15	0-1
Availability	0.15	0-1
Uptake_of_C_source	0.25	0-1
Maintenance	0.1	0-1
Radius-movement	0.15	0-1
Radius-action-predator	0.5	0-1
Immediate-killing?	Yes	Boolean
Aggressiveness	0.01-1.0	0-1
Max_viability_time	500	Positive float
Max_reproduction	50	Positive float
Min-mass		mass_reproduction/2
**Parameters of the medium**		
Diff-coeff	0-1	0-1
Max-occupation	2	Positive integer

REFERENCES

Ginovart, M., López, D., & Valls, J. (2002). INDISIM, an individual-based discrete simulation model to study bacterial cultures. Journal of Theoretical Biology, 214(2), 305–19. http://doi.org/10.1006/jtbi.2001.2466

Ginovart, M., & Prats, C. (2012). A bacterial individual-based virtual bioreactor to test handling protocols in a NetLogo platform. In I. Troch & F. Breitenecker (Eds.),

Proceedings of the 7th Vienna International Conference on Mathematical Modelling - MATHMOD 2012 Vienna Full Papers CD Volume (pp. 1495–1506). Wien: ARGESIM-ASIM German Simulation Society (ISBN: 978-3-901608-35-3). http://doi.org/10.3182/20120215-3-AT-3016.00115

Grimm, V., Berger, U., Bastiansen, F., Eliassen, S., Ginot, V., Giske, J., …etc… DeAngelis, D. L. (2006). A standard protocol for describing individual-based and agent-based models. Ecological Modelling, 198(1–2), 115–126. http://doi.org/10.1016/j.ecolmodel.2006.04.023

Grimm, V., Berger, U., DeAngelis, D. L., Polhill, J. G., Giske, J., & Railsback, S. F. (2010). The ODD protocol: A review and first update. Ecological Modelling, 221(23), 2760–2768. http://doi.org/10.1016/j.ecolmodel.2010.08.019
